# A new caddisfly genus (Trichoptera, Odontoceridae) from Vietnam

**DOI:** 10.3897/zookeys.65.489

**Published:** 2010-10-29

**Authors:** Tatiana I. Arefina-Armitage, Brian J. Armitage

**Affiliations:** Trichoptera, Inc., P.O. Box 21039, Columbus, Ohio 43221-0039 U.S.A.

**Keywords:** Caddisfly, Trichoptera, Odontoceridae, new genus, *Cephalopsyche*, new species, Vietnam

## Abstract

Cephalopsyche, a new genus of caddisfly (Trichoptera, Odontoceridae), is described from Vietnam. Two new species are placed in the genus: Cephalopsyche gorgona **sp. n.** and Cephalopsyche neboissi **sp. n.** The adult male and female of each species exhibit distinct sexual dimorphism, especially in head morphology. In males, there are hinged, chamber-like structures on the vertex of the head, containing filamentous, columnar tissue when exposed. Descriptions and illustrations of both species are provided.

## Introduction

The family Odontoceridae is relatively small and is known from all zoogeographical regions. Currently, the family is divided into two subfamilies: Odontocerinae, including 14 genera and Pseudogoerinae, with the monotypic genus Pseudogoera; three fossil genera are also known (Table 1). The family contains about 120 extant and fossil species and, except for Marilia and Psilotreta, most genera are regionally endemic, each containing 1–4 species, and are local in distribution.

**Table:** **Table 1.** World genera of Odontoceridae.

Genera by Biogeographic Regions	Number of Species	Distribution
**Afrotropical (Ethiopian)**		
Leptodermatopteryx Ulmer, 1910	1	Seychelles
**Australasian**		
Barynema Banks, 1939	2	Australia
**Indomalayan (Oriental)**		
Inthanopsyche Malicky, 1989	2	SE Asia
Lannapsyche Malicky, 1989	4	SE Asia
Phraepsyche Malicky & Chantaramongkol (in Malicky et al. 2000)	2	SE Asia
Cephalopsyche new genus	2	Vietnam
**Nearctic**		
Namamyia Banks, 1905	1	United States
Nerophilus Banks, 1899	1	United States
Parthina Denning, 1954	2	United States
· Phenacopsyche Cockerel, 1909	1	Miocene [United States]
Pseudogoera Carpenter, 1933	1	United States
**Neotropical**		
Anastomoneura Huamantinco & Nessimian, 2004	1	Brazil
Barypenthus Burmeister, 1839	1	Brazil
**Palaearctic**		
· Electrocerum Ulmer, 1912	1	Eocene [Baltic amber]
· Electropsilotes Ulmer, 1912	1	Eocene [Baltic amber]
Odontocerum Leach, 1815	3	Europe
Perissoneura McLachlan, 1871	2	Japan
**Widespread**		
Marilia F Müller, 1880	55	Asia [China, Southeast Asia], Australia, North America, Central America, and South America
Psilotreta Banks, 1899	40	Asia [China, India, Japan, Korea, Nepal, Russian Far East, Southeast Asia] and North America

During the course of examining material from the American Museum of Natural History, we found adult male and female specimens of two new, related odontocerid species, collected in Malaise traps during the spring of 1999 along the slope of Mt. Ngoc Linh (830–1460 m altitude), Quang Nam Province, Vietnam. These two species appear to be separated in part by altitude, but do co-exist or overlap at 950 m elevation. Both species differ substantially from known genera of the family and are here assigned to a new genus, Cephalopsyche, in the subfamily Odontocerinae. Primary terms used are according to Schmid (1998). Type material is preserved in alcohol and deposited in the collection of the American Museum of National History, New York, NY, USA.

## Taxonomy

### Cephalopsyche gen. n.

urn:lsid:zoobank.org:act:BD18AFC2-2B4F-41AC-99C5-2566E400947B

**Figure F2:**
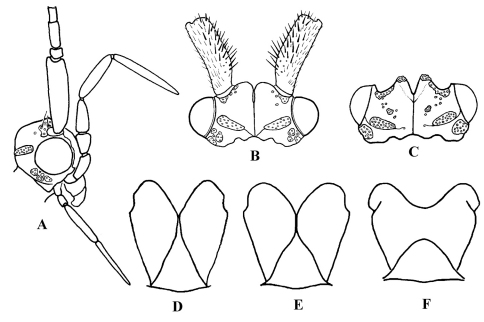
**Figure 2.** Cephalopsyche neboissi sp. n.: **A** male head, lateral **B** male head, dorsal **C** female head, dorsal and **D** male metathorax, dorsal. Psilotreta frigidaria Mey 1997: **E** – male metathorax, dorsal. Marilia sumatrana Ulmer 1951: **F** – male metathorax, dorsal.

**Figure F3:**
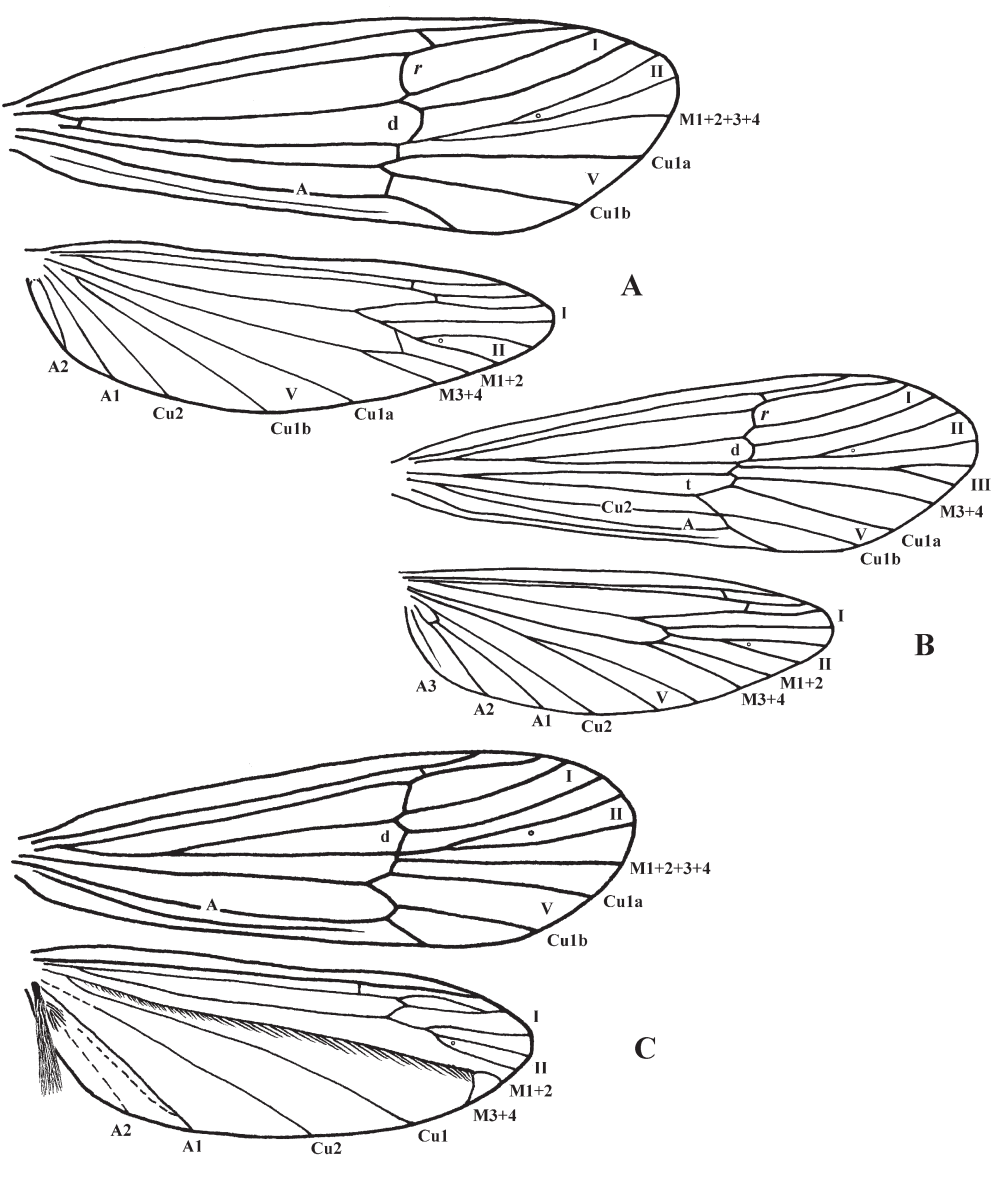
**Figure 3.** Wing venation. Cephalopsyche gorgona, sp. n.: **A** male **B** female. Cephalopsyche neboissi sp. n.: **C** – male.

**Figure F4:**
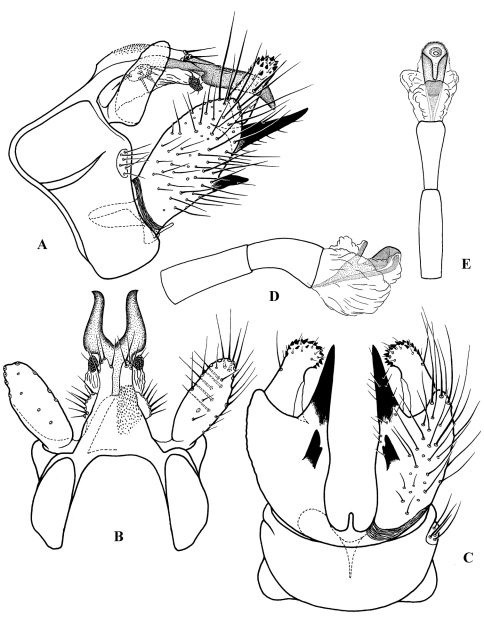
**Figure 4.** Cephalopsyche gorgona sp. n., male genitalia: **A** lateral **B** dorsal **C** ventral **D** phallus, lateral **E** phallus, dorsal.

**Figure F5:**
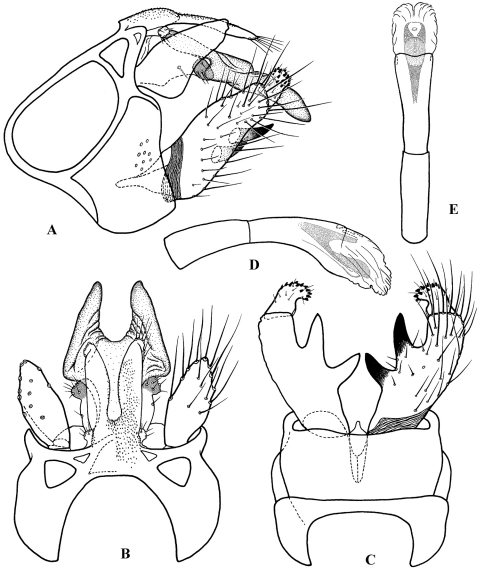
**Figure 5.** Cephalopsyche neboissi sp. n., male genitalia: **A** lateral **B** dorsal **C** ventral **D** phallus, lateral **E** phallus, dorsal.

Figs 1 [Fig F2] [Fig F3] [Fig F4] [Fig F5] 6 

#### Type species:

Cephalopsyche gorgona sp. n., original designation.

#### Other included species:

Cephalopsyche neboissi sp. n.

#### Diagnosis.

The new genus is distinguished from all Odontoceridae genera by combined characters of the head, wings, and genitalia. There are a few similarities with Marilia, particularly the absence of Cu2 in forewings of male; also, male of Cephalopsyche neboissi sp. n. bears a brush of long hairs on the anal lobe of the hind wings, typical for Marilia. In contrast, the hind wings in Cephalopsyche are similar in shape to the forewings, unlike the wider hind wings of Marilia, with an enlarged anal area.

**Figure:**
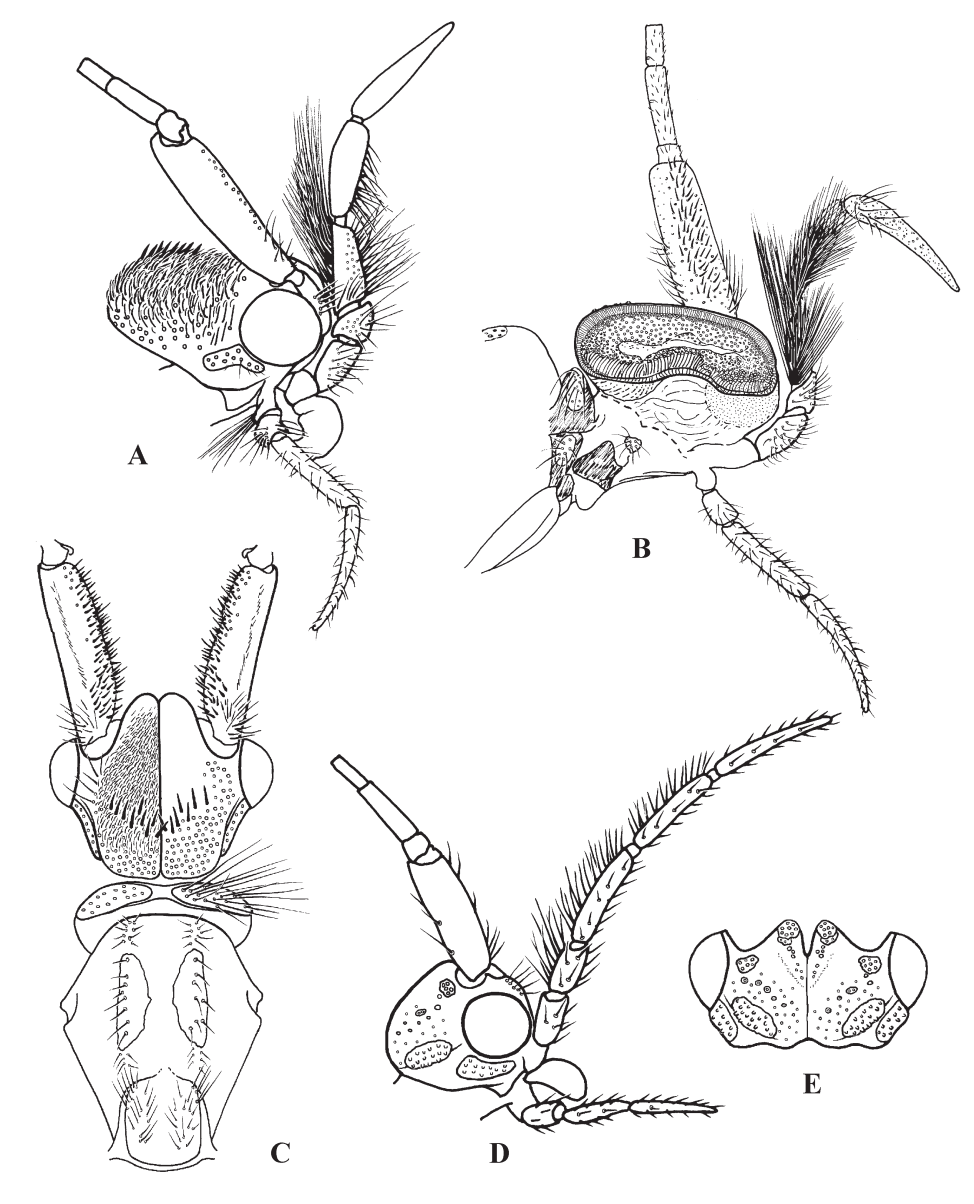
**Figure 1.** Cephalopsyche gorgona sp. n.: **A** male head, lateral **B** male head, lateral cross-section **C** male head and thorax, dorsal **D** female head, lateral **E** female head, dorsal.

Also, the new genus shares similarities in wing venation with the genera Lannapsyche Malicky, 1989 and Barynema Banks, 1939, namely the shape of the hind wings, nearly equal in width to forewings, and the presence of a transverse line of anastomosis (cord) in the male forewings. However, the male of Cephalopsyche differs by the presence of forks I, II, and V in both wings, versus forks I, II, III, and V in Lannapsyche and Barynema; also, it differs by fork II petiolate.

Finally, Cephalopsyche has several unique characters compared to all genera, including wing venation, specialized head structures, and genitalic features described below. However, based on the general structure of the male genitalia, it most closely resembles Psilotreta Banks, 1899. The following diagnosis emphasizes the comparison of these two genera [matching character states for Psilotreta in brackets] as a proxy for all other odontocerid genera. This new genus is unique when considering the following characters. Male with a “swollen” or domed head of varying extent [head not modified in this manner]. Scape thickened and longer than head, with spines along dorsomesal surface [subequal to length of head, lacks spines]. Eyes not enlarged, subequal in both sexes [male eyes larger than in female]. In male, fifth segment of maxillary palpi longest, tapered [fifth subequal to fourth or shorter, digitate]. Metathorax scutellum (Fig. 2D) triangular with short base and long, nearly straight sides [equilateral triangular with bent sides (Fig. 2E)]. Both pairs of wings in both sexes elongate and narrow, anal area in hind wings not enlarged [hind wings wider than forewings, anal area in hind wings enlarged]. Forewing crossvein *r* wide, lying near the base of fork I in both sexes, and is aligned with other crossveins in a transverse line of anastomosis [crossvein *r* halfway or more from the base of fork I to apical margin of forewing, no line of anastomosis]. Discoidal cell present only in forewings of both sexes [both pairs of wings with discoidal cell]. Discoidal cell long, joined for a very short distance by fork I [this distance much longer]. Cu2 in forewings of male absent [Cu2 present]. Anal cell missing in forewings of male [anal cell present]. Intermediate appendages of male genitalia lightly sclerotized with rounded, well sclerotized apical area possessing several setae [stout and sclerotized, in shape of curved or looped spurs]. Basal segment of inferior appendages of male genitalia bear a pair of large, stout spine-like, ventromesal processes [lacks spine-like processes]. Female with mesal plate of sternite IX well defined and more narrow than lateral lobes [sternite IX formed of sclerotized triangular or hemispherical plates, when present, of equal size].

##### Adult.

General color in alcohol yellowish-brown to brown, with vertex darker. Head displays distinct sexual dimorphism in shape and number of setal warts (Figs 1, 2). Frontal warts absent in male but present in female. Vertex of male head swollen or domed, movable, formed as a “chamber” lined with filamentous, columnar tissue. Head of female shorter than wide, anterior margin of vertex convex, with V-shaped mesal notch. Eyes of male and female not enlarged, nearly equal in size. Antennae slightly longer than forewings in both sexes. Scape thickened, slightly longer than head in both sexes, with spines on dorsomesal surface in male. Maxillary palpi 5-segmented and rather long in both sexes. Male maxillary palpi thick, heavily setose, with clusters of long, dense dark setae, mainly on third and fourth segments; second segment shorter than first and third; fourth longer than third; apical segment longest and tapered. Female maxillary palpi thinner but longer than in male, covered evenly with yellow to light brown setae; third, fourth, and fifth segments subequal, longer than first and second segments. Labial palpi 3-segmented in both sexes, longer in male than in female; first segment short, third slightly longer than second and tapered. Prothorax (Fig. 1C) with pair of large, elongate, transverse setal warts, bearing long setae in male, shorter setae in female. Mesothorax of male (Fig. 1C) with pair of large, oblong, longitudinal mesoscutal setal warts; almost twice shorter in female; mesoscutellar wart single. Metathorax scutellum (Fig. 2D) triangular with short base and long, nearly straight sides. Legs long and slender, foreleg shortest, mid leg and hind leg subequal; femur of mid leg as long as tibia; femur of hind leg nearly twice shorter than tibia. Tibial spur formula 2, 4, 4. Male and female wings elongate (Fig. 3), hind wings slightly narrower than forewings. Forks I, II, and V present in forewings of male, and I, II, III, and V in female; R1 meets wing margin near R2 in both sexes; crossvein *r* lies near base of fork I in both sexes, and aligned with other crossveins in a transverse line of anastomosis (cord); discoidal cell long, joined for very short distance by fork I; fork II petiolate; M without base (no thyridial cell) in male, represented by single apical branch originating from R4+5; in female M 3-branched, thyridial cell long and narrow; Cu2 absent in male; in female Cu2 ending at Cu1b; anal vein single in both sexes, no anal cell; postanal vein long. Venation of hind wings reduced; forks I, II, and V present in both sexes (fork V secondarily absent in male of Cephalopsyche neboissi); discoidal and thyridial cells absent; Sc and R1 run close to each other in female; two anal veins present in male, three in female; sparse long hairs along posterior edge of anal area.

##### Male genitalia

(Figs 4, 5): Segment IX stout and extended anteriorly in middle of lateral sides, with distinct setal area located posteriorly just above dorsal edge of inferior appendage. Dorsum of segment IX with paired, parallel lobes directed posterad. Preanal appendages large, elongate, earlike lobes in lateral view; elliptical in dorsal view. Segment X bifurcated, forming horn-like processes, with pair of setal, wart-like lobes located near base. Intermediate appendages lightly sclerotized with rounded, well sclerotized apical area possessing several setae. Basal segment of inferior appendages large, nearly 3 times as long as width at base in lateral view, having two large, stout, heavily sclerotized, spine-like ventromesal processes. Apical segment of inferior appendages short, straight in lateral view and slightly bent mesad in ventral view; bearing short, stout spines apically. Phallus with phallotheca sclerotized, long and cylindrical, slightly bent ventromesally; endotheca short and membranous; phallotremal sclerite large; parameres absent.

##### Female genitalia

(Fig. 6): Sternum VIII rectangular, with a row of setae along posterior margin. Segment IX stout. Sternite IX divided into three plates; mesal plate elongate, lying between lateral plates; each lateral plate forms fold posteriorly, which apparently receives spine-like ventromesal processes of male inferior appendages during copulation. Segment X very small, indistinguishably fused with segment IX. Appendages of segment X (Schmid 1998: 196), or setal warts of segment X (Parker and Wiggins 1987: 5) appressed, ovoid, slightly prominent in lateral view. Vaginal sclerites elongate, slightly sclerotized, and extending into segment VIII.

#### Etymology.

The genus name is a combination of the two Greek words: *cephalos* meaning head and *psyche* meaning soul.

#### Immature stages.

Unknown.

### Cephalopsyche gorgona sp. n.

urn:lsid:zoobank.org:act:9BD60E99-CD8E-4270-AC7A-97D9B5B4733E

Figs 1 3A–B 4 6A–B 

#### Holotype male:

**Vietnam:** Quang Nam Province, Ngoc Linh, 1460 m, 15°11.2'N, 108°2.3'E, Malaise trap, March – April 1999, D. Grimaldi, L. Herman, C. Johnson, K. Long, E. Sterling. **Paratypes:** 2 males, 1 female, same data as holotype; 1 male, 2 females, same locality as holotype, 30 March 1999, K. Long, C. Johnson; 1 female, ibid., 950 m, 15°10'N, 108°5'E, Malaise trap, 30 March 1999, K. Long, C. Johnson.

#### Diagnosis.

Male of Cephalopsyche gorgona differs from that of Cephalopsyche neboissi by the very large head, by the Minoan bull horn-like bifurcation of segment X in dorsal view, and by the unequal size and shape of the ventromesal processes of the inferior appendages. Female of this species can be easily distinguished in sternite IX by the shape of the posterior fold on each lateral plate and by the flask-shaped mesal plate.

##### Adult.

Male: forewings – 12.8–13.8 mm long, 4.0–4.4 mm wide; hind wings – 10.1–10.9 mm long, 2.9–3.2 mm wide. Female: forewings – 11.0–11.5 mm long, 3.4–3.5 mm wide; hind wings – 8.2–8.5 mm long, 2.6–2.7 mm wide. General color in alcohol yellow-brown to brown, with vertex darker. Thorax, lower portion of head, and legs of male brown to yellow-brown. Head of male with wart boundaries not distinct, posterior and antennal warts setal areas diffuse, frontal warts absent, posterolateral warts distinct, elongate, and subtending eyes. Head of male longer than wide; vertex heavily sclerotized, dark reddish-brown, abnormally enlarged, or swollen dorsoposteriorly (Fig. 1A–C); covered with dense, pale hairs, oriented mesoanterad, which appear appressed to head surface. Swollen portion of head formed as a “chamber” lined with filamentous, columnar tissue (Fig. 1B); chamber split into two hinged halves along fissure down middle of head oriented with body axis. Row of dark spines (7–11 on each side of head) forming transverse row across vertex of head. Head of female (Fig. 1D–E) with all setal warts distinct, frontal warts paired, antennal warts small and round, posterior warts elliptical, and posterolateral warts elongate. Male and female forewings elongate (Fig. 3A–B), brownish, with a few vague small lighter spots scattered mostly anteriorly; hind wings slightly narrower and paler. Forewing discoidal cell very long (extremely long in male, starting near base of wing).

##### Male genitalia

**** (Fig. 4): Segment IX stout, extended anteriorly slightly above midline of lateral sides. Dorsum of segment IX finely granular, subtriangular, with parallel lobes protruding posterad; lobes shorter than dorsum, finger-like, lightly sclerotized with a few long setae apically. Preanal appendages elongate, almost as long as maximum lateral width of segment IX, slightly broader subbasally and rounded apically in lateral view; almost oval in dorsal view, bearing scattered long, stout setae ventrally (Fig. 4B). Segment X well-developed, bifurcated, resembling Minoan bull horns, with acute tips pointed posterolaterad in dorsal view and posteroventrad in lateral view; a pair of oval, wart-like lobes located near base of segment X, each bearing 6–7 setae. Intermediate appendages extend slightly beyond lobes of segment IX dorsum, straight in dorsal view and slightly bent posteroventrally in lateral view; lightly sclerotized, each with rounded, well sclerotized apical area, possessing several setae. Basal segment of inferior appendages large, nearly elliptical in lateral view, 3 times as long as wide at base in lateral view, with two heavily sclerotized, spine-like, ventromesal processes; subapical process long, stout, equal in extent to apical segment of inferior appendages; second process short, with subapical notch in ventral view. Apical segment of inferior appendages short, straight in lateral view and slightly bent mesad in ventral view; bearing short, stout spines apically. Phallus with phallotheca sclerotized, long and cylindrical, bent ventromesally; endotheca short and membranous; phallotremal sclerite large; parameres absent.

**Figure:**
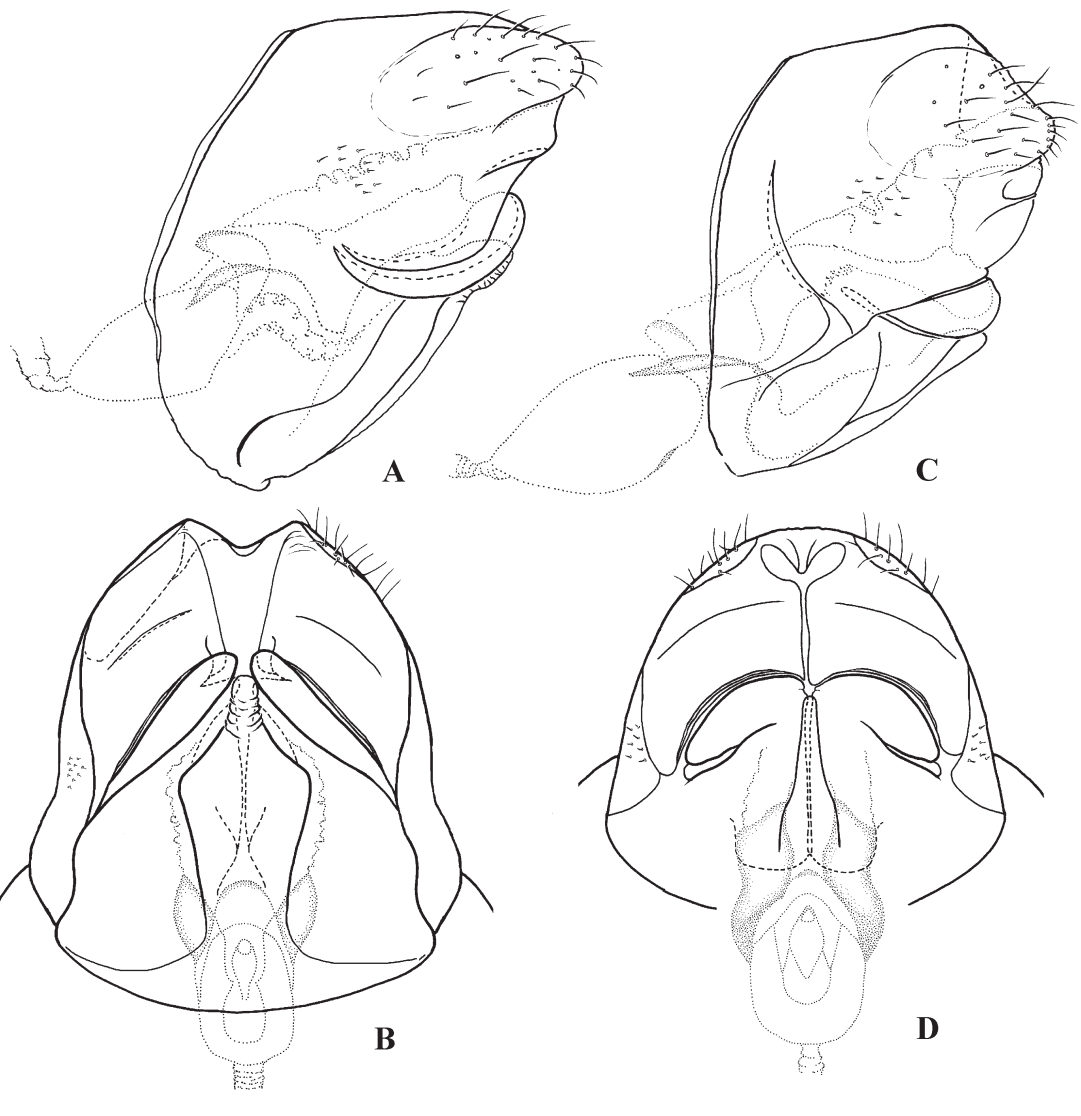
**Figure 6.** Female genitalia: Cephalopsyche gorgona sp. n.: **A** lateral **B** ventral; Cephalopsyche neboissi sp. n.: **C** lateral **D** ventral.

##### Female genitalia

(Fig. 6A–B): Mesal plate of sternite IX large, flask-shaped; each lateral plate of sternite IX forms elongate, diagonal fold posteriorly. In lateral view, segment X long, elongate, near elliptical in shape.

#### Distribution.

Known only from the type locality in Quang Nam Province (Vietnam).

#### Etymology.

This species was named for the Gorgons, three sisters of Greek mythology (who bore snakes on their heads and turned anyone who stared at them into stone) because of the mass of filamentous, columnar tissue found under the hinged vertex of the head.

### Cephalopsyche neboissi sp. n.

urn:lsid:zoobank.org:act:942CF862-BF10-4E4A-AA0A-CF7D699C960C

Figs 2A–D 3C 5 6C–D 

#### Holotype male:

**Vietnam:** Quang Nam Province, Ngoc Linh, 830 m, 15°10'N, 108°5'E, Malaise trap, 11–18 March 1999, K. Long, C. Johnson. **Paratypes:** 4 females, same data as holotype; 6 females, ibid., 950 m, 15°11.2'N, 108°2.3'E, Malaise trap, 16 April 1999, D. Grimaldi, L. Herman, C. Johnson, K. Long, E. Sterling.

#### Diagnosis.

Male of Cephalopsyche neboissi differs from that of Cephalopsyche gorgona by the smaller size of head, by the triangular shape of bifurcated branches of segment X in dorsal view, and by the subequal size and shape of the ventromesal processes of the inferior appendages. Female of this species can be distinguished in sternite IX by the narrow, diagonal pocket in the middle of each lateral plate and by the club-shaped mesal plate.

##### Adult.

Length of forewing: male – 11.6 mm; female – 10.3–11.5 mm. Male teneral, setal warts on head and thorax whitish, wings pale. Female coloration similar to female of Cephalopsyche gorgona. Head in both sexes shorter than wide, with frontal slit-shaped mesal notch in male and with V-shaped mesal notch in female. Male vertex of head reddish-brown, slightly swollen or domed and similar in structure to that of Cephalopsyche gorgona, but less enlarged. Antennal warts in both sexes small, elongate and subtend antennae; posterior warts elliptical and posterolateral warts elongate. Scapus of male with dorsomesal spines longer than in Cephalopsyche gorgona. Forewing venation of male resembles that of Cephalopsyche gorgona. Discoidal cell long, but shorter than intype species, situated in middle of wing. Venation of hind wings reduced and shifted to apex; forks I and II very short; fork V absent; M covered with golden bristles and forked near apex; brush of long hairs on anal lobe similar to Marilia (Schmid 1980, p. 295, fig. 845); wing membrane at A1 folded to hold brush when wings are at rest. Cluster of short hairs at base of A2. Wing venation of female very similar to Cephalopsyche gorgona.

##### Male genitalia

(Fig. 5): Segment IX stout, distinctly extended anteriorly near midline of lateral sides, with light-colored setal area posterolaterally. Dorsum of segment IX short, bilobed posteriorly, finely granular; lobes longer than length of dorsum, sclerotized and blade-like, with a few long setae apically. Preanal appendages large, shorter than maximal width of segment IX, elongate, broad subbasally, evenly tapering apically in lateral view, nearly elliptical in dorsal view. Segment X bifurcated, branches nearly triangular, with apices pointed posterad in dorsal view, and slightly posteroventrad in lateral view; pair of small, rounded setose lobes at base of segment. Intermediate appendage much shorter than lobes of segment IX dorsum, lightly sclerotized, each with rounded, well sclerotized apical area, bearing several setae. Basal segment of inferior appendages large, subrectangular, bearing two heavily sclerotized, horn-like ventromesal processes, similar in size and shape. Apical segment of inferior appendages short, with small, stout apical spines, slightly bent mesad in ventral view. Phallus with phallotheca sclerotized, long and cylindrical, slightly bent ventromesally; endotheca short and membranous; phallotremal sclerite large; parameres absent.

##### Female genitalia

(Fig. 6C–D): Mesal plate of sternite IX long, club-shaped; each lateral plate of sternite IX forms rounded, folded edge posteriorly, and with narrow, diagonal pocket in middle of plate, presumably to hold one horn-like ventromesal processes of male inferior appendages. In lateral view, segment X more rounded in shape.

#### Distribution.

Known only from the type locality in Quang Nam Province (Vietnam).

#### Etymology.

This species is named after the late Dr. Arturs Neboiss, Victoria Museum, Australia in recognition of his lifetime work on caddisflies.

## Phylogenetic considerations

Monophyly of Cephalopsyche is supported by at least seven unique characters, compared with the proxy genus Psilotreta, which are identified above in the diagnosis for the genus. Included among these is the suite of characters involved with the movable halves of the vertex of the head, covering a chamber filled with distinct filamentous, columnar tissue, and the basal segment of inferior appendages with a pair of large, stout spine-like, ventromesal processes.

Monophyly of Psilotreta initially rested on Parker and Wiggins (1987) statement: “The adult is distinguished by having the separation of R2 and R3 markedly basad of the separation of R4 and R5.” Unfortunately, this is also true of their stated sister genus Odontocerum Leach, 1815 and the genus Inthanopsyche Malicky, 1989. Thus, this character cannot be used for establishing monophyly for Psilotreta. Similarly, Schmid (1998), provided a character for Psilotreta, separating it from Marilia, involving the long, narrow discoidal cell of both wings, that is “joined by fork I for long distance.” However, this is another way of saying the same thing Parker and Wiggins (1987) said. For either case, the discoidal cell in Cephalopsyche is only present in the forewings and is joined by fork I for only a short distance. In order to establish monophyly for Psilotreta, we must turn to crossvein *r*, which is half or more distant from fork I and does not form a cord, or transverse line of anastomosis, with the other crossveins and fork bases. In Cephalopsyche, crossvein *r* is near the base of fork I and, in the forewings, is part of a cord. Monophyly has not been established for all genera within the family Odontoceridae. This family should be rigorously examined at the generic level to establish clear separation of genera and a sound phylogeny.

Parker and Wiggins (1987) enumerated a number of character states separating the North American species of Psilotreta from the Asian species. Interestingly, Cephalopsyche, although Asian, shares some characters with the North American species (e.g., absence of parameres). We hypothesize that Cephalopsyche arose from the same or a closely related ancestor as Psilotreta. Whereas Psilotreta became more widespread and modified, Cephalopsyche remained in the ancestral range, retaining many plesiomorphic characters, but still evolving some synapomorphies, such as the suite of characters involving the vertex of the head and loss of parameres.

## Discussion

The two hinged halves of the chamber that modifies the dorsal portion of the head suggest some functional role such as pheromone detection, similar to the occipital sclerites present on certain species of Hydroptilidae (Roemhild 1980) and the eversible lobes on the maxillary palp of some species of Psilotreta and Goera. Additional investigations will be required to confirm this.

The discovery of this new genus in Vietnam suggests that many more new species, and perhaps new genera, of caddisflies remain undescribed in the region. More thorough collecting, including repeated sampling over entire flight seasons and sampling of all microhabitats, will undoubtedly increase the known diversity of caddisflies from this country.

## Supplementary Material

XML Treatment for Cephalopsyche

XML Treatment for Cephalopsyche gorgona

XML Treatment for Cephalopsyche neboissi
